# The Hidden Dangers of Counterfeit and Replica‐Like Endodontic Files: A Scoping Review of Current Evidence

**DOI:** 10.1111/adj.70031

**Published:** 2025-12-19

**Authors:** Felipe Immich, Gustavo Henrique Longen, Carolyne Silveira da Motta, Bruna Cavalcante Chaves de Araújo, Giampiero Rossi‐Fedele, Lucas Peixoto de Araújo

**Affiliations:** ^1^ School of Dentistry Federal University of Pelotas Pelotas Brazil; ^2^ School of Dentistry Catholic University of Pelotas Pelotas Brazil; ^3^ Department of Cariology, Restorative Sciences & Endodontics, School of Dentistry University of Michigan Ann Arbor Michigan USA; ^4^ Adelaide Dental School The University of Adelaide Adelaide South Australia Australia

**Keywords:** copy, counterfeit, endodontic files, fake, Ni‐Ti, replica, review

## Abstract

This scoping review mapped and critically appraised laboratory evidence on counterfeit and replica‐like nickel–titanium endodontic files, evaluating design, metallurgical composition, surface finishing, and mechanical performance to clarify clinical and regulatory implications. Comprehensive searches of major databases and key endodontic journals identified 17 in vitro studies; no clinical studies were found. Counterfeit instruments consistently exhibited design irregularities, surface defects, and altered metallurgical properties that reduced cyclic fatigue resistance and produced unpredictable mechanical behaviour, posing significant patient‐safety concerns. Replica‐like instruments showed heterogeneous performance, with some matching or rarely exceeding original files in specific tests but others demonstrating inferior flexibility or torsional resistance. Quality‐control standards were inconsistent or lacking. These findings demonstrate that deviations in alloy processing, phase transformation, and surface finishing compromise mechanical reliability and highlight the urgent need for stronger regulatory oversight and clinical validation to safeguard practitioners and patients.

## Clinical Relevance

Counterfeit and replica‐like nickel‐titanium files have entered the dental market, often indistinguishable from authentic brands but differing in metallurgy, design, and performance. Laboratory evidence demonstrates that counterfeit instruments show poor manufacturing quality and unsafe mechanical behaviour, posing a significant risk of instrument fracture and treatment failure. Replica‐like files display variable properties, some comparable to originals and others inferior, highlighting the need for cautious selection and regulatory oversight.

## 1 Introduction

Since their introduction, nickel‐titanium (Ni‐Ti) rotary instruments have become essential in endodontic practice. Manufactured under standardised protocols, these tools are globally distributed by established companies [1]. Today, more than 200 Ni‐Ti rotary systems are available for root canal preparation [2].

High demand and cost have fuelled a market for cheaper alternatives [3]. Some companies previously uninvolved in endodontics now produce rotary instruments, often with inconsistent quality, occasionally replicating designs from established brands [2].

Low‐cost alternatives fall into two main categories: replica‐like and counterfeit systems [1]. Replica‐like systems aim to mimic well‐known brands by copying numbering, colour coding, and using names that closely resemble those of the original products [4]. They can be legally produced in some regions, as patent protections vary [5]. Counterfeit systems, on the other hand, are falsely marketed as authentic, with packaging designed to deceive buyers into thinking they are purchasing genuine products [6].

Authentic Ni‐Ti instruments are produced under strict quality control to ensure safety and performance [3]. While some replica‐like instruments may undergo manufacturer testing and have legal information available online [5], replica‐like and counterfeit products are often not manufactured to the same standards, which partly explains their lower cost [3, 4]. This problem is especially common in regions with limited import regulations or weak market oversight [1].

Counterfeit instruments are typically poor in quality and pose significant clinical risks [3, 6, 7]. Their use may lead to complications such as instrument fracture or canal transportation [3, 6]. With limited scientific data, dental professionals may be unaware of these products' safety or effectiveness [4].

Although the awareness is growing, the full impact of counterfeit and replica‐like instruments remains underreported [8]. There is a clear need to consolidate current evidence, identify knowledge gaps, and assess potential clinical consequences.

This scoping review aims to systematically map the available laboratory evidence on counterfeit and replica‐like Ni‐Ti endodontic files. Specifically, it seeks to characterise differences in design, metallurgical properties, surface finishing, and mechanical performance relative to original instruments, to explore the potential clinical implications of these variations, and to inform the endodontic community about the associated risks of using such instruments.

## 2 Methods

### 2.1 Protocol Registration

This scoping review protocol was pre‐registered on the Open Science Framework (https://osf.io/jbkm6/) to ensure transparency and methodological rigour. The review followed the Preferred Reporting Items for Systematic Reviews and Meta‐Analyses extension for Scoping Reviews (PRISMA‐ScR) guidelines [9].

### 2.2 Eligibility and Exclusion Criteria

Studies were included if they evaluated the mechanical, physical, or metallurgical properties of counterfeit or replica‐like endodontic files. Both in vitro and in vivo studies were eligible. No restrictions were placed on publication date, language, or study design.

Studies were excluded if they did not distinguish between counterfeit and replica‐like files. Reviews, case reports, conference abstracts, and editorials were also excluded.

### 2.3 Search Strategy

A comprehensive search was conducted by two reviewers (FI and LPA) across four databases: PubMed, Embase, Web of Science, and Scopus. The strategy combined Medical Subject Headings (MeSH) and free‐text terms related to counterfeit and replica‐like endodontic instruments (Supporting Information File 1). A manual search of the last 20 years of the International Endodontic Journal and Journal of Endodontics was also performed. The final search was completed on August 15, 2025.

### 2.4 Selection Process

Duplicate records were removed using Mendeley (Elsevier, Amsterdam, NE). Two independent reviewers screened titles and abstracts via the Rayyan online platform (Qatar Computing Research Institute, Doha, QA), 3w to each other's selections to minimise bias. Disagreements were resolved through discussion or consultation with a third reviewer.

Full texts of potentially eligible studies were retrieved and assessed against inclusion and exclusion criteria by the same reviewers. Reference lists of included articles were also screened for additional relevant studies.

### 2.5 Data Collection Process

Two reviewers independently extracted data using a standardised Excel spreadsheet (Microsoft Corporation, Redmond, WA, USA). Extracted information included: author(s), publication year, journal, study objectives, file type (original, counterfeit, replica‐like), acquisition source, tests performed, methods, sample size, and main findings. A third reviewer verified the data for accuracy.

### 2.6 Data Synthesis and Presentation

Extracted data were descriptively synthesised. Studies were grouped by file type (counterfeit or replica‐like) and evaluated properties. A narrative synthesis was conducted to identify common findings, trends, and inconsistencies.

Results are presented in narrative and tabular formats. Tables summarise key study characteristics, methodologies, and findings, offering an overview of current evidence on counterfeit and replica‐like endodontic Ni‐Ti instruments.

## 3 Results

### 3.1 Study Selection

**FIGURE 1:**
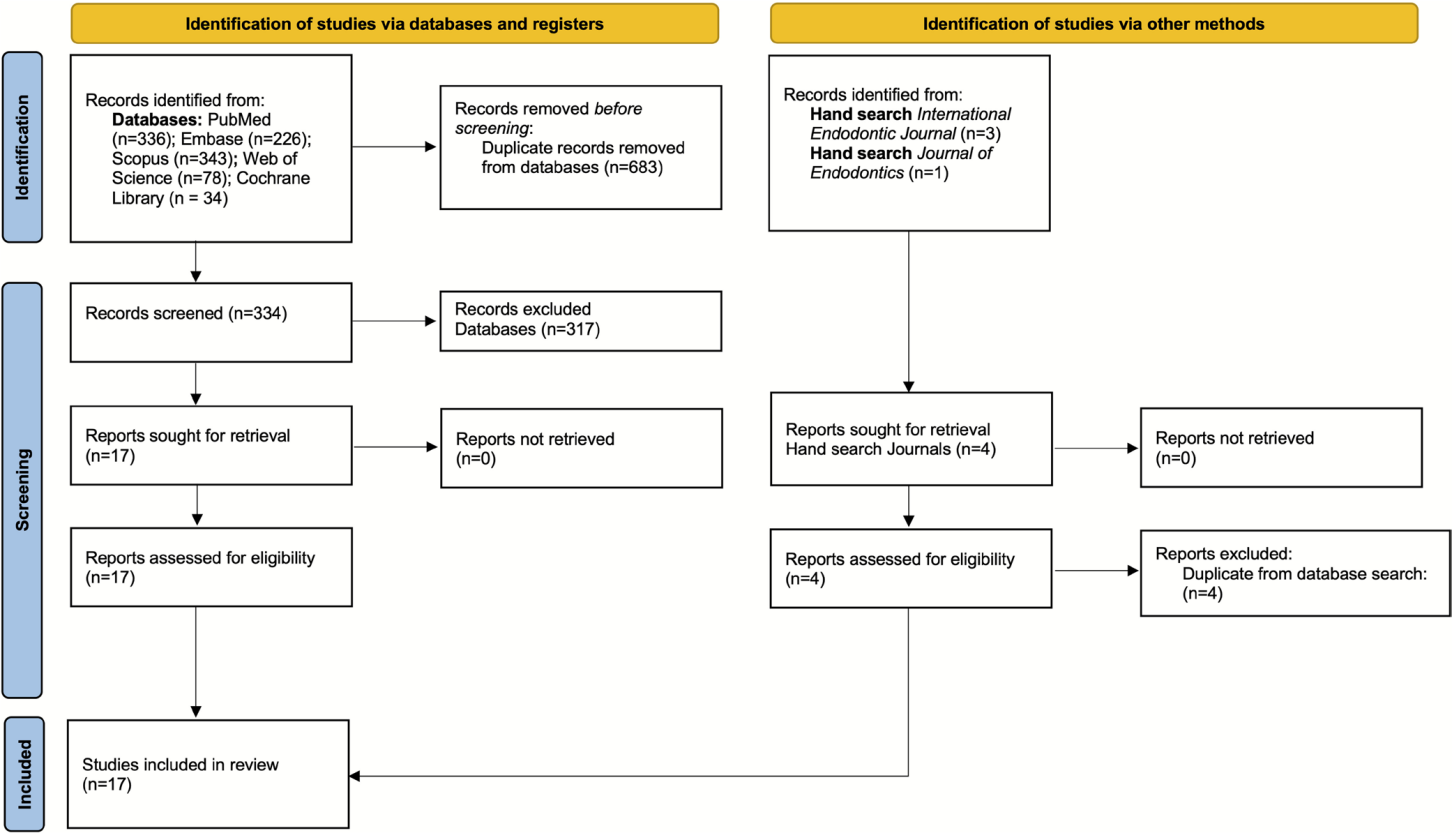
PRISMA 2020 flow diagram.

The initial database search yielded 1017 records. After removing duplicates, 334 articles remained. Title and abstract screening narrowed these to 17 studies for full‐text review. All 17 met the inclusion criteria and were included in the scoping review. The study selection process is detailed in the PRISMA flow diagram (Figure 1).

### 3.2 Characteristics of Included Studies

The 17 included studies investigated counterfeit and/or replica‐like endodontic Ni‐Ti files, focusing on their mechanical, physical, and metallurgical properties. Most were in vitro analyses involving mechanical testing, metallurgical evaluation, and design/surface characterisation. Details for counterfeit and replica‐like files are summarised in Tables 1 and 2, respectively.

**TABLE 1:** Evaluated properties, methods and main findings of studies on counterfeit endodontic files.

Author (Year)	Journal	Objectives	Files evaluated	Acquisition source of counterfeit files	Tests/Methods	Sample size	Main findings (instrument design)	Main findings (metallurgical characterisation)	Main findings (surface finishing)	Main findings (mechanical properties)
Ertas et al. 2014 [10]	BioMedical Engineering OnLine	To compare the CF resistance of original and counterfeit rotary instruments	ProTaper Universal files (Dentsply, Maillefer, Ballaigues, Switzerland)	NA	Design by SEM. Mechanical performance by CF	40 (20/group)	SEM analysis revealed that the cross‐section of the counterfeit file differed from that of the original	NA	NA	Original instruments had greater cyclic fatigue resistance than counterfeit ones. Counterfeit instruments displayed shallower dimples, indicating reduced plastic deformation
Madytianos et al. 2023 [3]	Australian Dental Journal	To evaluate and test differences in various physical and manufacturing properties of genuine and counterfeit rotary Ni‐Ti endodontic instruments	ProTaper Next. Mtwo	Dental supply company in China via the online purchasing platform Alibaba.com	Design and surface finishing by visual assessment and SEM. Metallurgical characterisation by EDX. Mechanical performance by microhardness and CF	96 (48/group)	The black length marking rings on original ProTaper Next instruments were precise, whereas they appeared faded in counterfeits	No significant differences in metallurgical composition between genuine and counterfeit ProTaper Next and Mtwo instruments, with nearly identical nickel and titanium proportions	Counterfeit instruments were less polished, with blunt tips and poorly machined surfaces. Original instruments had sharp, clean blades, while counterfeits exhibited defects, debris, and rolled edges	Original ProTaper Next had higher microhardness and lower variance than counterfeits; Mtwo showed similar hardness but higher variance in counterfeits. Both originals endured more cycles to fracture
Martins et al. 2021 [1]	International Endodontic Journal	Multimethod evaluation of design, metallurgy, mechanical performance, and shaping ability of two alternative ProTaper Next instruments: one replica‐like and one counterfeit	ProTaper Next (Dentsply Sirona, Ballaigues, Switzerland). X‐File (Denmark, Ludhiana, India)	Dental supply company in China via the online purchasing platform www.aliexpress.com.	Design and surface finishing by stereomicroscopic visual inspection. Metallurgical characterisation by DSC and EDS. Mechanical performance by CF, torsional and bending tests	359.	Similar blade number and helical angle, with no major defects. Counterfeit ProTaper Next had misaligned measuring lines and displayed a short coronal radial land absent in the originals. Tip geometry and blade transition angles also varied	All files were nearly equiatomic Ni‐Ti composition, with no other metals. Counterfeit ProTaper Next had similar phase transformation temperatures to originals but with high variability	Original instruments had horizontal grinding marks, while counterfeits exhibited irregular surface finishes	Counterfeits had lower cyclic fatigue resistance, higher fracture angles, and reduced bending flexibility, except X3; maximum torque was similar
Martins et al. 2022 [11]	Materials	To evaluate the overall design, metallurgical properties, and mechanical performance of original and counterfeit ProGlider instruments	ProGlider (Dentsply Sirona, Ballaigues, Switzerland)	Dental supply company in China via the online purchasing platform www.aliexpress.com	Design and surface finishing by SEM and stereomicroscopy. Metallurgical characterisation by DSC and EDX analysis. Mechanical performance by CF, torsional and bending resistance tests.	70 (35/group)	Both had similar blades and cross‐sections, but counterfeits had misaligned measuring lines, different colour coding/markings, and rounded tips versus sharp originals	Both instruments were nearly equiatomic Ni‐Ti with no other metals. Heat treatment was present in both. At 20°C, originals were mixed austenite/R‐phase, while counterfeits were fully austenitic	Both had grinding marks, but counterfeits showed additional irregularities and microdefects, including metal rollovers on blade edges	Original had a longer time to fracture and greater flexibility, with similar fragment length and maximum torque; it also showed a higher angle of rotation
Noenko et al. 2023 [12]	Journal of International Dental and Medical Research	Comparison of SEM features of original and counterfeit SOCO SC Plus files to identify potential markers for detecting falsified instruments	SOCO SC Pro (Chengdu Sani Medical Equipment Co., Chengdu China)	Internet‐market	Design and surface finishing by SEM	90 (45/group)	Counterfeit files had a larger tip base, sharper tip and residual compounds at the shank, indicating a pasting assembly technique	NA	Counterfeit had a higher prevalence of scrapings and cracks	NA
Rodrigues et al. 2017 [6]	International Endodontic Journal	Visual and mechanical comparison of original vs. counterfeit Reciproc R25	Reciproc R25 (VDW, Munich, Germany)	Internet via the online purchasing platform www.mercadolivre.com.br	Design by direct observation, stereomicroscopy and SEM. Surface finishing by roughness test. Metallurgical characterisation by EDX. Mechanical performance by bending resistance test, CF and Vickers microhardness test	70 (35/group)	Counterfeits had inconsistent colour coding, misaligned measurement lines, a pink stopper with one cut‐out, a thicker spade‐shaped tip, less defined edges, and shallower flutes	Original files used equiatomic Ni‐Ti, while counterfeits were nickel‐rich Ni‐Ti	Original instruments were smoother than counterfeits	Originals had lower bending resistance and microhardness but longer fatigue life; all showed ductile fracture with dimples and fatigue striations, and similar fracture sites
Unal et al. 2026 [13]	Microscopy Research and Technique	To compare the design, mechanical, and metallurgical properties of the original, replica‐like, and two counterfeit file systems	Reciproc Blue R25 (VDW, Munich, Germany). Recip‐One Files Blue R25 (Rogin Dental, Shenzhen, China)	Via the online purchasing platform www.aliexpress.com	Design by visual inspection, photographs, stereomicroscopy and SEM. Mechanical performance by CF. Metallurgical characterisation by DSC and energy‐dispersive spectroscopy	100 (25/group)	Counterfeit files had irregular markings, pale pink stoppers, and cutting tips; counterfeit file 2 had distinct packaging, file 1 lower taper, and both were darker blue compared with the original	Ni‐Ti content was similar; originals were austenitic at body temperature, while counterfeits were martensitic	Scraping marks and surface irregularities were observed in the cutting regions of the counterfeit files	No significant difference was observed between the systems for time to fracture

Abbreviations: CF, cyclic fatigue; DSC, differential scanning calorimetry; EDX, energy dispersive x‐ray spectroscopy; NA, not available; Ni‐Ti, nickel‐titanium; SEM, scanning electron microscopy.

**TABLE 2:** Evaluated properties, methods, and main findings of studies on replica‐like endodontic files.

Author (Year)	Journal	Objectives	Original files	Replica‐like files	Tests/Methods	Sample size	Main findings (instrument design)	Main findings (metallurgical characterisation)	Main findings (surface finishing)	Main findings (mechanical properties)
Alnoury et al. 2024 [14]	Saudi Endodontic Journal	To compare CF resistance of three replica systems vs. ProTaper Gold	ProTaper Gold (Dentsply Maillefer, Ballaigues, Switzerland)	MG3 Gold (Perfect Medical Instruments Co. Ltd., Shenzhen, China). V TAPER Gold (Fanta Dental Co. Ltd., Shanghai, China)	Design by SEM. Mechanical performance by CF	45 (15/group)	V TAPER Gold Fanta had a triangular cross‐section; ProTaper Gold, a convex triangle; MG3 Gold Perfect, a less convex triangle	NA	NA	V TAPER Gold Fanta lasted nearly 3× longer and withstood more cycles than ProTaper Gold and MG3 Gold Perfect, which performed similarly
Aydin et al. 2024 [15]	Applied Bionics and Biomechanics	Evaluate CF resistance of three replica systems vs. ProTaper Next	ProTaper Next (Dentsply‐Sirona, Ballegiues, Switzerland)	EndoArt Touch Gold (InciDental, İstanbul, Turkey). Perfect MTF Plus Gold (Perfect Medical Instruments, Shenzhen, China). Fanta V‐Taper Gold (Fanta Dental, Shenzhen, China)	Design by SEM. Mechanical performance by CF, Topographic analysis of the fractured surfaces with SEM	60 (15/group)	Most instruments matched the manufacturer's cross‐section, except EndoArt Touch Gold, which was irregularly rectangular or round	NA	NA	ProTaper Next and Perfect MTF Plus Gold have significantly higher number of cycles to failure compared to EndoArt Touch Gold and Fanta V‐Taper Gold
Bastos et al. 2024 [16]	Australian Endodontic Journal	To compare CF, torsional fatigue, bending, and buckling resistance a one replica system vs. Reciproc Blue	Reciproc Blue (VDW, Munich, Germany)	One File Blue (Denco, Shenzhen, China)	Mechanical performance by CF, Torsional and flexural resistance, and maximum buckling load	80 (40/group)	NA	NA	NA	Reciproc Blue and Only One File Blue had similar fatigue resistance and fragment length; Reciproc Blue had higher torque, rotation angle, and buckling resistance, while Only One File Blue was more flexible
Martins et al. 2020 [4]	Journal of Endodontics	Evaluation of cyclic fatigue resistance and metal alloy properties of three replica rotary instruments originals under continuous rotation and optimum torque reverse (OTR) motion	ProTaper Universal (Dentsply Maillefer, Ballaigues, Switzerland). ProTaper Gold (Dentsply Maillefer, Ballaigues, Switzerland)	U‐File (Dentmark, Ludhiana, India). Super Files (Shenzhen Flydent Medical, Shenzhen, China). Super Files Blue (Shenzhen Flydent Medical)	Metallurgical characterisation by EDX and DSC. Mechanical performance by CF (Instruments were activated with a continuous clockwise rotation or optimum torque reverse function)	100 (20/group) (10/motion)	NA	All instruments had similar Ni‐Ti ratios; replicas had higher austenitic transformation temperatures, with ProTaper Universal lowest and Super Files Blue highest	NA	All systems lasted longer in optimum torque reverse motion than in continuous rotation; replicas outperformed originals in both motions
Martins et al. 2021 [1]	International Endodontic Journal	Multi‐method assessment of design, metallurgy, mechanical performance, and shaping ability of one replica‐like and one counterfeit ProTaper Next instrument	ProTaper Next (Dentsply Sirona, Ballaigues, Switzerland)	X‐File (Dentmark, Ludhiana, India)	Design by stereomicroscopic visual inspection. Metallurgical characterisation by DSC and EDX. Mechanical performance by CF, torsional and bending tests	359.	Instruments were generally similar in blade number, helical angle, and defect‐free design; X‐File X3 had fewer blades, shorter cutting area, misaligned lines, a short radial land, rounded edges. Tip geometry and transition angles also varied among instruments	Both had similar Ni‐Ti ratios with no other metals; X‐File had the lowest phase transformation temperature, ProTaper Next the highest with greatest R‐phase finish	ProTaper Next showed horizontal grinding marks, while X‐File instruments had a smoother surface	X‐File and ProTaper Next had similar cyclic fatigue, torque, and bending resistance; X‐File X2 showed different fracture angles versus ProTaper Next X2.
Martins et al. 2021 [17]	Revista Portuguesa de Estomatologia	Multimethod comparison of design, metallurgy, torsional strength, and flexibility of ProTaper Gold SX and replica‐like instruments	ProTaper Gold SX instrument (Dentsply Maillefer, Ballaigues, Switzerland)	Premium Taper Gold (Waldent, City not stated, China). Go‐Taper Flex (Access, Shenzhen, China)	Design and surface finishing by stereomicroscopic visual inspection. Metallurgical characterisation by EDX/SEM and DSC. Mechanical performance by torsional and bending tests	30 (10/group)	The three SX instruments were similar in blade count, helix angle, and cross‐section, with no defects; Go‐Taper Flex differed with a flat‐tip‐like geometry	All instruments were nearly equiatomic Ni‐Ti with no other metals; Premium Taper Gold showed mixed austenite/R‐phase, while ProTaper Gold and Go‐Taper Flex had similar, more martensitic transformation temperatures	ProTaper Gold showed machining marks, Premium Taper Gold was smoother, and Go‐Taper Flex had the most irregular surface	ProTaper Gold and Go‐Taper Flex differed in maximum torque; angles of rotation were similar across instruments, while Go‐Taper Flex was less flexible than ProTaper and Premium Taper Gold
Ragozzini et al. 2024 [18]	International Journal of Dentistry	Evaluation of autoclave sterilisation effects on four replica‐like instruments versus WaveOne Gold, including fracture number and static cyclic fatigue performance	WaveOne Gold (Dentsply Sirona, Ballaigues, Switzerland)	TF4‐Gold (Shenzhen Perfect Medical Instruments, Shenzhen, China). Roll‐Wave‐Gold (Shenzhen Denco Medical, Shenzhen, China) W‐File (TDK, Shenzhen, China). Micro‐Gold (Microdont, Shenzhen, China)	Design and surface finishing by SEM. Mechanical performance by CF	50 (10/group)	All instruments had non‐cutting tips; WaveOne Gold's tips were more rounded, others triangular	NA	WaveOne Gold had a well‐finished surface; TF4 Gold, Roll Wave Gold, W File, and Micro Gold were less refined, with minor defects like porosities in WaveOne Gold	WaveOne Gold, TF4 Gold, and Wave Roll Gold had similar cyclic fatigue resistance, higher than W File and Micro Gold, which also differed from each other. Sterilisation had no effect; distortions appeared after the third use in TF4 Gold and W File, and after the first three uses in Roll Wave Gold and Micro Gold
Reis et al. 2023 [19]	Scientific Reports	To evaluate the effect of the number of uses and autoclave sterilisation on the cyclic fatigue resistance of Reciproc Blue and four replica‐like endodontic instruments	Reciproc Blue (VDW, Munich, Germany)	RC Blue (Shenzhen Perfect Medical Instruments, Shenzhen, China). Only One File Blue (Shenzhen Denco Medical, Shenzhen, China). Recip One Blue (Shenzhen Rogin Medical, Shenzhen, China). Micro Blue (Microdont, Shenzhen, China)	Design and surface finishing by SEM. Mechanical performance by CF	50 (10/group)	All instruments had non‐cutting tips; RC Blue and Recip One Blue were more rounded; Only One File Blue and Micro Blue were triangular	NA	Reciproc Blue had good initial finishing but deformed after three uses; RC Blue, Only One File Blue, Recip One Blue, and Micro Blue were less refined, with minor defects observed in Reciproc Blue	Fracture times were similar, except for Micro Blue, which was lower. Sterilisation had no effect; deformations occurred in RC Blue and One Blue after the third use, only One File Blue after all uses, and none in Micro Blue
Ríos‐Osorio et al. 2025 [20]	Journal of Clinical and Experimental Dentistry	To compare the cyclic fatigue resistance of original and replica‐like reciprocating endodontic files, following steam sterilisation cycles and/or immersion in 5% sodium hypochlorite	Reciproc Blue (VDW, Munich, Germany). One Reci (Micro‐Mega, Besancon, France). R‐Motion (FKG Dentaire SA, La Chaux‐de‐Fonds, Switzerland)	Roll Wave Gold (Shenzhen Denco Medical, Shenzhen, China). RCS Blue T (RAMO Medical, Suzhou, China)	Metallurgical composition by EDX. Mechanical performance by CF	105 (15/group)	NA	Reciproc Blue had higher carbon and oxygen than One Reci, higher carbon than R‐Motion and RCS Blue T, and higher oxygen than Roll Wave; R‐Motion had the highest oxygen overall	NA	Reciproc Blue had the highest cyclic fatigue resistance, followed by R‐Motion, One Reci, Roll Wave, and RCS Blue T (lowest). R‐Motion, One Reci, and Roll Wave did not differ. Fatigue resistance decreased in Reciproc Blue, R‐Motion, and One Reci after three autoclave and NaOCl cycles
Tarragó et al. 2025 [5]	European Endodontic Journal	Comparison of cyclic fatigue resistance in single and double curvatures of reciprocating replica‐like versus original instruments, and evaluation of tip sizes against manufacturer specifications	Reciproc R25 (VDW, Munich, Germany). Reciproc Blue R25 (VDW, Munich, Germany)	Reverso Silver (Access, Paris, France—manufactured by Shenzen SuperLine technology, Shenzen, China). Reverso Blue (Access, Paris, France—manufactured by Shenzen SuperLine technology, Shenzen, China)	Design by measuring tip size with a digital calliper. Mechanical performance by CF	72 (18/group)	The tip sizes of all instruments were smaller than the manufacturer's stated value and fell outside the expected range	NA	NA	Reciproc Blue and Reverso Blue fractured later than Reverso Silver; in single curvatures, they also outlasted Reciproc. Double curvatures shortened fracture times, except for Reciproc
Unal et al. 2025 [13]	Microscopy Research and Technique	To compare the design, mechanical, and metallurgical properties of original, replica‐like, and counterfeit file systems	Reciproc Blue R25 (VDW, Munich, Germany)	Recip‐One Blue R25 (Rogin Dental, Shenzhen, China)	Design by visual inspection, photographs, stereomicroscopy and SEM. Metallurgical characterisation by DSC and EDX. Mechanical performance by CF	100 (25/group)	Recip‐One blades were darker blue than Reciproc Blue; both had similar tip diameter, taper, and passive tip designs	Ni‐Ti content was similar; Reciproc Blue was austenitic at body temperature while Recip‐One was martensitic	NA	No statistically significant difference was observed between the systems for time to fracture
Uslu et al. 2022 [21]	Australian Endodontic Journal	To evaluate the design and standardisation, CF resistance, torsional resistance, composition, and phase transformation of replica‐like files of ProTaper Next and Reciproc systems, compared with those of the original brand files	ProTaper Next (Dentsply Sirona, Ballaigues, Switzerland). Reciproc (VDW Munich, Germany)	X File (NIC Shenzhen Superline Technology, Shenzhen, China). Only One File (Shenzhen Denco Medical, Shenzhen, China)	Design by stereomicroscopy and SEM. Metallurgical characterisation by EDX. Mechanical Performance by CF and torsional resistance	144 (72 original, 72 replica‐like).	Only One File had a larger tip than ProTaper Next, X File, and Reciproc; X File had a higher taper than ProTaper Next, with Reciproc and Only One File similar. Replica‐like systems had active tips, no deformities, and measurement line deviations—greater in Only One File and X File	Only One File had higher Ni content than ProTaper Next and X File; all files showed single‐step transformations, with ProTaper Next and Reciproc having lower martensite‐to‐austenite temperatures, while X File and Only One File were near body temperature	NA	Reciproc had the highest cyclic fatigue and torsional resistance; Only One File had higher fatigue but lower torsional resistance than ProTaper Next and X File, which were similar. Rotation angles did not differ

Abbreviations: CF, cyclic fatigue; DSC, differential scanning calorimetry; EDX, energy dispersive x‐ray spectroscopy; NA, not available; Ni‐Ti, nickel‐titanium; SEM, scanning electron microscopy.

### 3.3 Counterfeit Files

A total of six studies evaluated counterfeit files by comparing them with their genuine counterparts. Counterfeit file systems included ProTaper Universal [10], Mtwo [3], ProTaper Next [1, 3], ProGlider [11], SOCO SC Pro [12], Reciproc [6, 13], and Reciproc Blue [13]. Most counterfeit instruments were sourced from e‐commerce platforms (e.g., Alibaba, Aliexpress, MercadoLivre), though some origins were unspecified.

#### 3.3.1 Instrument Design

Comparisons between original files and their counterfeit counterparts revealed multiple design discrepancies across ProTaper, Reciproc, and other rotary systems. For ProTaper Universal, Scanning Electron Microscopy (SEM) analysis showed that the cross‐section of counterfeit files differed from the originals [10]. For ProTaper Next, black length marking rings were precise in genuine instruments but appeared faded in counterfeits, and misaligned measurement lines (> 0.1 mm) were observed [3]. Counterfeit ProTaper Next X3 files additionally displayed a short radial land on the coronal portion of active blades, which was absent in originals, and tip geometry and transition angles varied between counterfeit and original instruments [1, 3].

Counterfeit Reciproc and Reciproc Blue files showed inconsistent ISO colour coding, irregular or missing measurement lines, and altered stoppers. The originals feature smooth red stoppers with three cut‐outs, whereas the counterfeits have pink stoppers with only one [6, 13]. Tips of counterfeit files were thicker, eccentric, or spade‐shaped, in contrast to the original non‐cutting tips [6]. Reciproc Blue counterfeits exhibited darker blue colouration, pale pink stoppers not following the ISO coding of the shank, irregular measurement markings, and, in some cases, lower taper values [13]. Original Reciproc instruments had S‐shaped cross‐sections with sharp cutting edges, whereas counterfeit files had less defined edges and shallower, smaller flutes [6].

Similarly, ProGlider counterfeits maintained the same number of blades and helical angles as the originals, with symmetrical blade geometry and square cross‐sections, but measuring lines were misaligned by 0.7 mm, and the tip was rounded instead of sharp; colour‐coding and marking designs also differed [11]. SOCO SC Plus counterfeit files exhibited a larger working tip base, sharper tip design, and residual compounds at the shank fixation area, suggesting the use of a pasting technique for assembling rotary instrument parts [12].

Overall, counterfeit files across multiple systems consistently showed deviations in tip design, cross‐sectional geometry, measurement markings, and colour coding, reflecting significant departures from the standardised design of the original instruments.

#### 3.3.2 Metallurgical Characterisation

Analyses of the metallurgical composition revealed differences between original and counterfeit files across systems. ProTaper Next, Mtwo, and its counterfeit counterparts exhibited nearly identical nickel‐titanium ratios, with no other metallic elements detected; Differential Scanning Calorimetry (DSC) testing showed that counterfeit instruments had phase transformation temperatures close to the originals, though with greater variability [1, 3]. Similarly, ProGlider originals and counterfeits had nearly equiatomic Ni‐Ti compositions, but DSC testing indicated differences in phase behaviour: at room temperature, the original displayed a mixed austenite and R‐phase structure, while the counterfeit was fully austenitic, suggesting altered heat treatment [11]. In contrast, counterfeit Reciproc files differed in composition from the originals, being machined from Ni‐Ti alloy wire enriched in nickel, whereas originals used equiatomic Ni‐Ti [6]. For Reciproc Blue, Ni‐Ti levels were similar, but the original files had an austenitic finish temperature (Af) of 33.97°C, below body temperature, indicating an austenitic structure, while the counterfeit systems had Af values above 37°C, consistent with a martensitic structure at body temperature [13].

#### 3.3.3 Surface Finishing

Surface analyses consistently demonstrated inferior finishing in counterfeit files across multiple systems. SEM examination of ProTaper Next and Mtwo counterfeits revealed less polished surfaces, blunt cutting tips, rolled edges, and debris, whereas originals had sharp, well‐machined blades [1, 3]. ProGlider counterfeits exhibited similar grinding marks to originals but included additional irregularities and microdefects, such as metal rollovers on blade edges [11]. SOCO SC Plus counterfeit files presented a higher prevalence of scrapings and cracks compared with the originals, suggesting compromised surface integrity [12]. Reciproc and Reciproc Blue counterfeits also showed notable surface defects, with higher roughness and scraping marks in the cutting regions, while the original instruments maintained smoother, more uniform surfaces [6, 13]. Overall, counterfeit files consistently demonstrated poorer surface finishing, which may affect cutting efficiency and clinical performance.

#### 3.3.4 Mechanical Properties

Mechanical testing consistently demonstrated superior performance of original instruments compared to counterfeit files across multiple systems, although some exceptions were noted. ProTaper Universal originals exhibited significantly higher cyclic fatigue resistance than counterfeits, with SEM revealing deeper dimples at the fracture plane, indicative of greater plastic deformation, while counterfeits displayed shallower dimples [10]. Genuine ProTaper Next instruments had higher microhardness and lower variance than counterfeits, and both ProTaper Next and Mtwo originals withstood significantly more cycles to fracture [3]. Counterfeit ProTaper Next instruments had lower cyclic fatigue resistance and reduced flexibility in bending tests, although maximum torque and maximum bending load were similar to the originals [1].

The original ProGlider showed a higher mean time to fracture and greater flexibility than its counterfeit counterpart, although fragment length and maximum torsional load were similar; the original also exhibited a higher angle of rotation [11]. Original Reciproc instruments had a longer cyclic fatigue life despite lower bending resistance and microhardness compared to counterfeits, with SEM revealing ductile fracture characteristics, fatigue striations, and no plastic deformation in the helical shaft, consistent with flexural fatigue failure [6]. For Reciproc Blue, no statistically significant differences were observed between original and counterfeit files in time to fracture [13]. Overall, while most original files outperformed counterfeit instruments in fatigue resistance, flexibility, and fracture behaviour, exceptions exist depending on the specific file system and test parameter.

### 3.4 Replica‐Like Files

A total of eleven studies evaluated replica‐like files by comparing them with their original counterparts. These studies investigated various brands of replica instruments, including MG3 Gold, V‐TAPER Gold, MG EndoArt Touch Gold, Perfect MTF Plus Gold, U‐File, Super Files, Premium Taper Gold, Go‐Taper Flex, TF4‐Gold, Roll Wave Gold, W‐File, Micro‐Gold, One File Blue, RCS Blue T, RC Blue, Recip One Blue, Micro Blue, X File, Only One File, Reverso Silver, and Reverso Blue. The original files used for comparison included ProTaper Universal, ProTaper Next, ProTaper Gold, ProTaper Gold SX, WaveOne Gold, Reciproc, and Reciproc Blue [1, 4, 5, 13, 14, 15, 16, 17, 18, 19, 20, 21].

#### 3.4.1 Instrument Design

Design analyses revealed both similarities and differences between ProTaper systems and their replicas. ProTaper Gold displayed a convex triangular cross‐section, while V‐Taper Gold Fanta had a triangular form and MG3 Gold Perfect a less convex triangular shape [14]. ProTaper Next, Perfect MTF Plus Gold, and V‐Taper Gold largely matched their manufacturers' descriptions, whereas EndoArt Touch Gold showed irregular rectangular or round cross‐sections [15]. Comparisons of ProTaper Next and X‐File demonstrated general similarity in blade number and helical angle, but X‐File X3 presented fewer blades, a shorter cutting area, misaligned markings, and rounded cutting edges, differing from the sharp edges of ProTaper Next [1]. ProTaper Gold SX and Premium Taper Gold had comparable overall designs, though Go‐Taper Flex exhibited a flat tip configuration distinct from the originals [17]. Only One File showed a significantly larger tip diameter than both ProTaper Next and X‐File, and X‐File had a higher taper than ProTaper Next. Replica‐like files generally featured active tips, unlike the passive design of the originals, and demonstrated greater deviations in measurement lines [21].

Several differences were highlighted between the original Reciproc systems and replicas. SEM evaluations showed that most instruments (Reciproc Blue, RC Blue, Recip One Blue, Only One File Blue, and Micro Blue) featured non‐cutting tips, although tip morphology varied, with RC Blue and Recip One Blue exhibiting more rounded tips, while Only One File Blue and Micro Blue presented triangular‐shaped tips [19]. In contrast, in another study, Only One File displayed a significantly larger tip diameter than Reciproc, with no taper differences detected between them. SEM observations also revealed that the replica‐like system had an active tip design, unlike the original Reciproc files, and showed greater deviations in measurement lines [21]. Tip size assessments revealed that Reciproc Blue, Reciproc, Reverso Silver, and Reverso Blue all had values smaller than the manufacturer's specification (0.25 mm) and outside the expected tolerance range, with significant differences among groups [5]. The Recip One Blue system showed tip diameter and taper values similar to Reciproc Blue, both with passive tip designs [13]. SEM analysis of WaveOne Gold files and their replicas demonstrated non‐cutting tips across all instruments, with WaveOne Gold exhibiting a more rounded tip design, whereas TF4‐Gold and Roll‐Wave‐Gold showed triangular tips [18].

#### 3.4.2 Metallurgical Characterisation

Metallurgical analyses showed that Ni–Ti ratios were similar across Reciproc, Reciproc Blue, and replica files [13, 20, 21]. DSC testing revealed single‐step transformations during heating and cooling in all files, with Reciproc exhibiting higher martensite‐to‐austenite transformation temperatures than X File and Only One File, closer to body temperature [21]. Reciproc Blue displayed a higher weight percentage (wt%) of carbon and oxygen compared with One Reci, higher carbon than R‐Motion and RCS Blue T, and higher oxygen than Roll Wave, while R‐Motion showed the highest oxygen content overall [20]. Transformation behaviour differed among systems: Reciproc Blue had an Af value of 33.97°C, below body temperature, consistent with an austenitic structure, whereas Recip One Blue exhibited Af values above 37°C, indicating a martensitic structure at body temperature [13].

All ProTaper systems and their replicas exhibited a nearly equiatomic Ni–Ti composition with no other metallic elements detected [4, 21]. Replica instruments—including U‐File, Super Files, Super Files Blue, X‐File, Premium Taper Gold, Go‐Taper Flex, and Only One File—generally had higher austenitic transformation temperatures than the original files. ProTaper Universal showed the lowest transformation temperature, while Super Files Blue and ProTaper Next had the highest, with ProTaper Next also showing greater R‐phase content [1, 4]. DSC analyses indicated mixed austenite and R‐phase in Premium Taper Gold, whereas ProTaper Gold SX and Go‐Taper Flex exhibited more martensitic characteristics [17]. Only One File had a higher Ni weight percentage than ProTaper Next and X File, and transformation temperatures of ProTaper Next occurred closer to body temperature compared with X File and Only One File [1, 21].

#### 3.4.3 Surface Finishing

SEM analysis revealed differences in surface finishing between original files and replica instruments. ProTaper Next displayed horizontal grinding marks, whereas the replica X‐File exhibited a smoother surface [1]. ProTaper Gold SX showed visible machining marks, Premium Taper Gold had a smoother surface with fewer irregularities, and Go‐Taper Flex presented the most irregular surface appearance [17]. WaveOne Gold exhibited a satisfactory initial finish, while TF4 Gold, Roll Wave Gold, W File, and Micro Gold showed less refined surfaces; minor defects such as porosities in cutting edges and flutes were observed in the WaveOne Gold group [18]. Replica instruments, including RC Blue, Only One File Blue, Recip One Blue, and Micro Blue, had lower surface quality, with Reciproc Blue showing only minor defects such as porosities in cutting edges and flutes [19].

#### 3.4.4 Mechanical Properties

Cyclic fatigue resistance varied among the systems. In cyclic fatigue testing, Reciproc Blue outperformed most replica files but showed similar performance to Recip One Blue, RC Blue, and Only One File Blue; RCS Blue T consistently showed the lowest resistance [16, 20, 21]. Regarding other mechanical properties, Reciproc Blue exhibited superior torque to fracture, greater rotation angle, and higher buckling resistance than Only One File Blue, which in turn showed greater flexibility [16].

Reciproc Blue and Reverso Blue fractured later than Reverso Silver and Reciproc in single curvatures, while Reverso Silver fractured earliest under double curvature; overall, fracture times were shorter under double curvature, except for Reciproc, which showed no significant difference [5]. In torsional resistance, Reciproc showed the highest values, Only One File the lowest, and proximal angles of rotation were similar across all systems [21]. WaveOne Gold, TF4 Gold, and Roll Wave Gold performed similarly, whereas W File and Micro Gold had significantly lower values [18]. Regarding distortion and deformation, TF4 Gold and W File exhibited distortions after three uses, whereas Roll Wave Gold and Micro Gold were affected after all uses [18].

After autoclave sterilisation, cyclic fatigue decreased in R‐Motion and One Reci after three cycles of autoclaving and 5% sodium hypochlorite immersion compared with controls [20]. Sterilisation and sodium hypochlorite exposure reduced cyclic fatigue resistance in Reciproc Blue and One Reci but did not alter fracture incidence across groups, though replicas such as RC Blue, Recip One Blue, and Only One File Blue displayed more frequent deformations after repeated use [19, 20].

Mechanical testing of ProTaper systems and their replica‐like counterparts revealed variable performance across different properties. In cyclic fatigue resistance, V‐TAPER Gold exhibited approximately three times the time to failure and number of cycles to failure compared with ProTaper Gold and MG3 Gold Perfect, which performed similarly [14]. ProTaper Next and Perfect MTF Plus Gold showed higher cycles to failure than EndoArt Touch Gold and Fanta V‐Taper Gold, while Only One File had the highest values, exceeding ProTaper Next and X File, which were comparable [15, 21]. Under torsional stress, ProTaper Next, ProTaper Gold SX, and X File demonstrated similar maximum torque to fracture, whereas Only One File exhibited the lowest torsional resistance. Replica instruments such as Go‐Taper Flex showed lower flexibility and reduced maximum bending load compared with ProTaper Gold SX and Premium Taper Gold [17, 21]. Fracture angle and rotation were largely similar across systems, except X‐File X2, which differed from ProTaper Next X2 [1]. Additionally, all systems showed significantly longer time to fracture under optimum torque reverse motion compared with continuous rotation, with some replicas outperforming their original counterparts in both motions, including ProTaper Universal and ProTaper Gold [4].

## 4 Discussion

The primary aim of this scoping review was to systematically map laboratory evidence on the performance of counterfeit and replica‐like Ni‐Ti endodontic files. Peer‐reviewed clinical data are nearly absent, as using counterfeit files in patients is ethically impermissible. Laboratory findings, however, raise significant safety concerns. In contrast, the clinical use of replica‐like files may be acceptable under specific conditions, particularly in resource‐limited regions or medical institutions, where low‐cost instruments can offer a cost‐effective alternative—provided they are obtained through legitimate channels and meet basic quality standards. Despite the limited evidence, replica‐like endodontic files are widely used worldwide.

Counterfeit instruments are typically of inferior quality and illegally mimic original brands, often sold at significantly lower prices [6, 11]. A report by the Commission on the Theft of American Intellectual Property indicated that China and Hong Kong were responsible for 87% of counterfeit goods seized upon entering the United States [22]. Online sales have accelerated global access to these products, with all reviewed studies citing e‐commerce platforms as acquisition sources [1, 3, 6, 11, 12, 13]. Among those specifying, most identified China as the manufacturing country.

Replica‐like files are legally produced and marketed, often by companies in emerging economies, capitalising on lower production costs and expanding markets [4]. Most reviewed studies reported replica‐like files originating from manufacturers in China [4, 5, 13, 14, 15, 16, 17, 18, 19, 20, 21] and India [1, 4]. Despite their legal status, no international regulatory frameworks currently mandate standardised quality control for Ni‐Ti rotary or reciprocating systems [4]. Consequently, clinicians may inadvertently use devices lacking sufficient scientific validation, exposing patients to potential risks [19].

Counterfeit files often visually resemble authentic ones but exhibit subtle design inconsistencies that can undermine clinical performance. While blade number and helical angle are generally similar, flaws such as misaligned measuring lines, altered tip geometry, different taper, and faded colour coding have been reported [1, 3, 6, 11, 13]. Examples include rounded or spade‐shaped tips and shallow flutes that may reduce cutting efficiency and increase the risk of canal blockage or fracture [23]. Additionally, some counterfeit files featured active cutting tips, unlike the non‐cutting tips of the originals [6, 13], which may increase the risk of apical transportation [24, 25, 26], particularly in curved canals and if the operator is unaware of the design differences. End‐users should identify and avoid using counterfeit instruments.

Replica endodontic files generally resemble the overall design of original systems, but often differ in key features such as tip morphology, cutting edge sharpness, taper, and measurement markings [1, 5, 14, 15, 17, 18, 19, 21]. These variations may lead replicas to behave differently from their originals, potentially altering cutting efficiency, flexibility, and clinical performance [23]. Such unpredictability is compounded by the limited number of in vitro *studies* evaluating their behaviour, highlighting a need for further research to understand their safety and efficacy in practice. Two studies revealed that replica files displayed geometry and tip designs inconsistent with manufacturer specifications, raising concerns about their reliability [5, 15]. Importantly, such discrepancies were not limited to replicas, as even original Reciproc and Reciproc Blue files were found to have smaller tip sizes than those declared by the manufacturer [5], underscoring potential risks for clinical performance and quality control.

Metallurgical analyses revealed that counterfeit files often fail to replicate the proprietary thermal and structural features of original instruments, despite showing broadly similar Ni‐Ti ratios. In some systems, such as ProTaper Next, Mtwo, and ProGlider, counterfeits exhibited nearly equiatomic Ni‐Ti compositions and phase transformation temperatures approximating the originals, but with greater variability, suggesting inconsistencies in alloy processing and heat treatment [1, 3, 11]. Such variability raises concerns, as small deviations in transformation behaviour can significantly alter flexibility and fatigue resistance [27, 28, 29, 30]. More strikingly, counterfeit Reciproc files showed clear compositional differences, being manufactured from Ni‐Ti wire enriched in nickel, unlike the equiatomic alloy of the originals [6], which could increase stiffness [31]. Even when compositions were similar, as in Reciproc Blue, transformation temperatures diverged: the originals demonstrated an austenitic structure at body temperature, while the counterfeits remained martensitic, which could drastically alter their clinical behaviour and performance [13]. These discrepancies indicate that counterfeits not only lack the precise thermal treatments defining original performance but also possess fundamentally different phase transformation properties, compromising predictability and raising serious concerns about their safety, durability, and clinical reliability.

Replica files also frequently mirrored the overall Ni‐Ti ratios of original systems; however, subtle yet clinically relevant differences emerged in their metallurgical profiles. DSC tests revealed that Reciproc exhibited higher martensite‐to‐austenite transformation temperatures than X File and Only One File, aligning more closely with body temperature, which may favour stability under clinical conditions [13]. In contrast, Reciproc Blue showed an Af value below body temperature, consistent with an austenitic structure, while Recip One Blue exhibited Af values above 37°C, implying a martensitic structure during use, which would increase flexibility [13]. Such divergent transformation behaviours indicate that replicas, despite similar compositions, may respond very differently under functional stresses. Furthermore, elemental analyses also revealed compositional variability, with Reciproc Blue showing higher levels of carbon and oxygen than several replicas, while R‐Motion exhibited the highest oxygen content overall [20]. Elevated concentrations of these elements are associated with a greater proportion of martensitic crystalline structure [20, 32], which can enhance alloy flexibility and fracture resistance [20]. Together, these differences in phase transformation behaviour and composition indicate that replicas, despite similar Ni‐Ti ratios, cannot be assumed equivalent to originals, making their mechanical performance unpredictable.

Surface finishing is a key determinant of cyclic fatigue resistance, as well‐polished, defect‐free surfaces significantly improve instrument durability [33, 34, 35]. Counterfeit endodontic files consistently exhibited inferior surface finishing compared with originals, with SEM analyses revealing blunt tips, rolled edges, debris, irregular grinding marks, and microdefects across multiple systems [1, 3, 6, 11, 12, 13]. In contrast, original instruments showed sharp, well‐polished, and uniformly machined surfaces. These defects in counterfeits are clinically significant, as surface irregularities increase stress concentration, reduce cutting efficiency, and accelerate crack initiation, ultimately compromising both performance and safety [34, 36].

SEM analyses of original and replica files revealed that original files generally exhibited well‐controlled machining patterns, with ProTaper Next, ProTaper Gold SX, and WaveOne Gold showing sharp, uniform surfaces and Reciproc Blue displaying only minor porosities, while most replicas exhibited surface defects, porosities, and irregularities in cutting edges and flutes [17, 18, 19]. Notably, the X‐File and Premium Taper Gold replicas showed a smoother, well‐polished surface compared to the originals, which may enhance fatigue resistance [1, 17]. Overall, while isolated replicas may achieve favourable finishing, the widespread surface defects in most replica files can reduce cutting efficiency, increase stress concentration, and elevate fracture risk, highlighting serious concerns about their clinical reliability and safety [34, 36].

Cyclic fatigue, a surrogate measure of instrument separation [37], generally revealed superior performance of original instruments compared with counterfeits, though exceptions occurred. ProTaper Universal, ProTaper Next, Mtwo, and ProGlider originals showed higher fatigue resistance, flexibility, and microhardness, with SEM analyses confirming more favourable fracture patterns, whereas counterfeits exhibited shallower dimples, reduced flexibility, and shorter fatigue lives [1, 3, 10, 11]. Reciproc files similarly outperformed counterfeits, displaying longer fatigue life and ductile fracture features [6]. By contrast, Reciproc Blue and its counterfeits showed no significant difference in time to fracture [13], likely due to the martensitic structure of counterfeits at body temperature, reduced taper and tip diameters, and artificial canal conditions that lowered file‐to‐canal stress [6, 13, 36, 38, 39].

Overall, while originals usually demonstrate superior mechanical reliability, testing conditions may obscure clinically relevant weaknesses of counterfeit instruments [13], reinforcing their unpredictable and unsafe nature. Even in cases where counterfeit files appear to perform similarly [13], equivalent results cannot be assumed, as these products are not consistently manufactured nor subject to standardised quality control [3, 6, 7]. Their evaluation is confined to laboratory settings—clinical trials would be unethical—and outcomes such as cyclic fatigue, although widely used, remain only surrogate indicators of instrument fracture with limited clinical translation [37].

Across studies comparing replica files, mechanical performance was heterogeneous and protocol‐dependent, with originals often performing best, but several replicas matched or even outperformed them under specific conditions. In cyclic fatigue, Reciproc Blue was superior to many replicas, but performed similarly to Recip One Blue, RC Blue, Recip One Blue, and Only One File Blue [13, 16, 19], likely due to the martensitic phase of some replicas at body temperature versus the austenitic phase of Reciproc Blue [13]. Additionally, Reciproc Blue exceeded Only One File Blue in torque, rotation angle, and buckling resistance, whereas the replica showed greater flexibility [16]. Reciproc Blue and Reverso Blue (replica) outlasted Reverso Silver and Reciproc under various curvatures [5], reflecting the fatigue‐optimising effects of blue thermomechanical treatment [40]. WaveOne Gold performed similarly to TF4 Gold and Roll Wave Gold, whereas W File and Micro Gold were inferior [18].

Regarding ProTaper‐family systems, V‐Taper Gold Fanta (replica) displayed nearly three times longer fatigue life than ProTaper Gold and MG3 Gold Perfect [14], while in another study, ProTaper Next and Perfect MTF Plus Gold (replicas) outperformed EndoArt Touch Gold and Fanta V‐Taper Gold [15]. Only One File showed the highest fatigue resistance, surpassing ProTaper Next and equalling X‐File, but this came at the cost of lower torsional resistance [21]—a predictable outcome given its reciprocating kinematics, which improves cyclic fatigue relative to rotary files [37]. ProTaper Next and X‐File performed similarly in torque to fracture [21]. Notably, replicas including U‐File, Super Files, and Super Files Blue exhibited significantly longer time to fracture than their originals (ProTaper Universal and ProTaper Gold) [4]. This was hypothesised to result from differences in transformation temperatures, giving the replicas a martensitic structure at body temperature, in contrast to the austenitic structure of the originals, which increased flexibility and cyclic fatigue resistance [4]. Overall, while originals typically provide more predictable mechanical performance, several replicas demonstrated comparable or superior properties in isolated tests, highlighting their occasionally competitive behaviour.

The findings of this scoping review provide valuable insights into the laboratory performance of counterfeit and replica‐like Ni‐Ti endodontic files. Counterfeit files should be avoided altogether due to their inferior quality, design inconsistencies, poor surface finishing, metallurgical irregularities, and unpredictable mechanical performance, all of which substantially increase the risk of instrument fracture and compromise clinical safety. Regarding replica files, although they replicate original instruments and may closely resemble them, laboratory studies have identified important differences in design features, surface finishing, phase transformation temperatures, and mechanical behaviour. These discrepancies indicate that replica files should not be assumed to perform equivalently to their original counterparts. Nonetheless, the performance of certain replica files is encouraging, suggesting they may serve as a viable alternative in resource‐limited settings. However, their evaluation has been limited to laboratory studies, which have restricted clinical translation, whereas extensive literature supports the efficacy and safety of the original files. Clinical studies are therefore necessary to robustly assess the effectiveness and safety of replica files before they can be recommended for routine use.

## 5 Conclusion

This scoping review highlights that counterfeit Ni‐Ti endodontic files pose serious safety risks and should be strictly avoided due to consistent design flaws, poor surface finishing, metallurgical inconsistencies, and unpredictable mechanical performance. Replica files, while legally produced and sometimes demonstrating promising laboratory performance, exhibit notable variations in design, surface quality, phase transformation behaviour, and mechanical properties compared with original instruments, indicating that equivalent clinical performance should not be assumed. Although some replica files may offer a cost‐effective alternative in resource‐limited settings, their current evaluation is limited to in vitro studies with restricted clinical applicability. Therefore, rigorous clinical investigations are essential to determine the safety and efficacy of replica files and to guide their responsible use in endodontic practice.

## Author Contributions

**Felipe Immich:** conceptualization, investigation, writing – original draft, methodology, validation, software, formal analysis. **Lucas Peixoto de Araújo:** conceptualization, investigation, writing – original draft, writing – review and editing, visualization, validation, methodology, formal analysis, project administration, supervision, data curation.

## Funding

The authors have nothing to report.

## Conflicts of Interest

The authors declare no conflicts of interest.

## Supporting information


**Supplementary File 1.** Search strategy.

## Data Availability

The data that support the findings of this study are openly available in the Open Science Framework at https://osf.io/jbkm6/.
