# Supplementary material for: The Hidden Dangers of Counterfeit and Replica‐Like Endodontic Files: A Scoping Review of Current Evidence
Source: Aust Dent J. 2025 Dec 19;71(1):5–25. doi: 10.1111/adj.70031 (PMC12945868; doi:10.1111/adj.70031)
Supplement: Supplementary file 1 — Search strategy. [file ADJ-71-5-s001.docx]

| **Supplementary File 1 –** Search strategies | | |
| --- | --- | --- |
|  | **Search Terms** | |
| ***Pubmed*** | | |
| #3 | Search #1 AND #2 | |
| #2 | Seach (Counterfeit) OR (Fake) OR (Falsified) OR (Forged) OR (Imitation) OR (Replica) OR (Replica-like) OR (Copy) |  |
| #1 | Search (Tooth, Nonvital) OR (Tooth, nonvital) OR (Nonvital Tooth) OR (Tooth, Devitalized) OR (Devitalized Tooth) OR (Tooth, Pulpless) OR (Pulpless Tooth) OR (Teeth, Pulpless) OR (Pulpless Teeth) OR (Teeth, Devitalized) OR (Devitalized Teeth) OR (Teeth, Nonvital) OR (Nonvital Teeth) OR (Teeth, Endodontically-Treated) OR (Endodontically-Treated Teeth) OR (Teeth, Endodontically Treated) OR (Tooth, Endodontically-Treated) OR (Endodontically-Treated Tooth) OR (Tooth, Endodontically Treated) OR (Root canal therapy) OR (Canal Therapies, Root) OR (Canal Therapy, Root) OR (Root Canal Therapies) OR (Therapies, Root Canal) OR (Therapy, Root Canal) OR (Endodontics) OR (Endodontics) OR (Endodontology) |  |
| ***Embase*** | | |
| #3 | Search #1 AND #2 | |
| #2 | Seach (Counterfeit) OR (Fake) OR (Falsified) OR (Forged) OR (Imitation) OR (Replica) OR (Replica-like) OR (Copy) | |
| #1 | Search (Tooth, Nonvital) OR (Tooth, nonvital) OR (Nonvital Tooth) OR (Tooth, Devitalized) OR (Devitalized Tooth) OR (Tooth, Pulpless) OR (Pulpless Tooth) OR (Teeth, Pulpless) OR (Pulpless Teeth) OR (Teeth, Devitalized) OR (Devitalized Teeth) OR (Teeth, Nonvital) OR (Nonvital Teeth) OR (Teeth, Endodontically-Treated) OR (Endodontically-Treated Teeth) OR (Teeth, Endodontically Treated) OR (Tooth, Endodontically-Treated) OR (Endodontically-Treated Tooth) OR (Tooth, Endodontically Treated) OR (Root canal therapy) OR (Canal Therapies, Root) OR (Canal Therapy, Root) OR (Root Canal Therapies) OR (Therapies, Root Canal) OR (Therapy, Root Canal) OR (Endodontics) OR (Endodontics) OR (Endodontology) | |
| ***Web of Science*** | | |
| #3 | Search #1 AND #2 | |
| #2 | TS=((Counterfeit) OR (Fake) OR (Falsified) OR (Forged) OR (Imitation) OR (Replica) OR (Replica-like) OR (Copy)) | |
| #1 | TS=((Tooth, Nonvital) OR (Tooth, nonvital) OR (Nonvital Tooth) OR (Tooth, Devitalized) OR (Devitalized Tooth) OR (Tooth, Pulpless) OR (Pulpless Tooth) OR (Teeth, Pulpless) OR (Pulpless Teeth) OR (Teeth, Devitalized) OR (Devitalized Teeth) OR (Teeth, Nonvital) OR (Nonvital Teeth) OR (Teeth, Endodontically-Treated) OR (Endodontically-Treated Teeth) OR (Teeth, Endodontically Treated) OR (Tooth, Endodontically-Treated) OR (Endodontically-Treated Tooth) OR (Tooth, Endodontically Treated) OR (Root Canal Therapy) OR (Canal Therapies, Root) OR (Canal Therapy, Root) OR (Root Canal Therapies) OR (Therapies, Root Canal) OR (Therapy, Root Canal) OR (Endodontics) OR (Endodontics) OR (Endodontology)) | |
| ***SciVerse Scopus*** | | |
| #3 | Search #1 AND #2 | |
| #2 | TITLE-ABS-KEY ( (“Counterfeit”) OR (“Fake”) OR (“Falsified”) OR (“Forged”) OR (“Imitation”) OR (“Replica”) OR (“Replica-like”) OR (“Copy”) ) | |
| #1 | TITLE-ABS-KEY ( (“Tooth, Nonvital”) OR (“Tooth, nonvital”) OR (“Nonvital Tooth”) OR (“Tooth, Devitalized”) OR (“Devitalized Tooth”) OR (“Tooth, Pulpless”) OR (“Pulpless Tooth”) OR (“Teeth, Pulpless”) OR (“Pulpless Teeth”) OR (“Teeth, Devitalized”) OR (“Devitalized Teeth”) OR (“Teeth, Nonvital”) OR (“Nonvital Teeth”) OR (“Teeth, Endodontically-Treated”) OR (“Endodontically-Treated Teeth”) OR (“Teeth, Endodontically Treated”) OR (“Tooth, Endodontically-Treated”) OR (“Endodontically-Treated Tooth”) OR (“Tooth, Endodontically Treated”) or (“Root Canal Therapy”) OR (“Canal Therapies, Root”) OR (“Canal Therapy, Root”) OR (“Root Canal Therapies”) OR (“Therapies, Root Canal”) OR (“Therapy, Root Canal”) OR (“Endodontics”) OR (“Endodontics”) OR (“Endodontology”) ) | |
| ***The Cochrane Library*** | | |
| #3 | Search #1 AND #2 | |
| #2 | TITLE-ABS-KEY (Counterfeit) OR (Fake) OR (Falsified) OR (Forged) OR (Imitation) OR (Replica) OR (Replica-like) OR (Copy) | |
| #1 | TITLE-ABS-KEY (Tooth, Nonvital) OR (Tooth, nonvital) OR (Nonvital Tooth) OR (Tooth, Devitalized) OR (Devitalized Tooth) OR (Tooth, Pulpless) OR (Pulpless Tooth) OR (Teeth, Pulpless) OR (Pulpless Teeth) OR (Teeth, Devitalized) OR (Devitalized Teeth) OR (Teeth, Nonvital) OR (Nonvital Teeth) OR (Teeth, Endodontically-Treated) OR (Endodontically-Treated Teeth) OR (Teeth, Endodontically Treated) OR (Tooth, Endodontically-Treated) OR (Endodontically-Treated Tooth) OR (Tooth, Endodontically Treated) OR (Root canal therapy) OR (Canal Therapies, Root) OR (Canal Therapy, Root) OR (Root Canal Therapies) OR (Therapies, Root Canal) OR (Therapy, Root Canal) OR (Endodontics) OR (Endodontics) OR (Endodontology) | |
